# Secreted Citrate Serves as Iron Carrier for the Marine Pathogen *Photobacterium damselae* subsp *damselae*

**DOI:** 10.3389/fcimb.2017.00361

**Published:** 2017-08-08

**Authors:** Miguel Balado, Beatriz Puentes, Lucía Couceiro, Juan C. Fuentes-Monteverde, Jaime Rodríguez, Carlos R. Osorio, Carlos Jiménez, Manuel L. Lemos

**Affiliations:** ^1^Department of Microbiology and Parasitology, Institute of Aquaculture, University of Santiago de Compostela Santiago de Compostela, Spain; ^2^Department of Chemistry, Faculty of Sciences and Center for Advanced Scientific Research (CICA), University of A Coruña A Coruña, Spain

**Keywords:** *Photobacterium damselae*, citrate, iron uptake, siderophores, vibrioferrin

## Abstract

*Photobacterium damselae* subsp *damselae* (*Pdd*) is a *Vibrionaceae* that has a wide pathogenic potential against many marine animals and also against humans. Some strains of this bacterium acquire iron through the siderophore vibrioferrin. However, there are virulent strains that do not produce vibrioferrin, but they still give a strong positive reaction in the CAS test for siderophore production. In an *in silico* search on the genome sequences of this type of strains we could not find any ORF which could be related to a siderophore system. To identify genes that could encode a siderophore-mediated iron acquisition system we used a mini-Tn*10* transposon random mutagenesis approach. From more than 1,400 mutants examined, we could isolate a mutant (BP53) that showed a strong CAS reaction independently of the iron levels of the medium. In this mutant the transposon was inserted into the *idh* gene, which encodes an isocitrate dehydrogenase that participates in the tricarboxylic acid cycle. The mutant did not show any growth impairment in rich or minimal media, but it accumulated a noticeable amount of citrate (around 7 mM) in the culture medium, irrespective of the iron levels. The parental strain accumulated citrate, but in an iron-regulated fashion, being citrate levels 5–6 times higher under iron restricted conditions. In addition, a null mutant deficient in citrate synthase showed an impairment for growth at high concentrations of iron chelators, and showed almost no reaction in the CAS test. Chemical analysis by liquid chromatography of the iron-restricted culture supernatants resulted in a CAS-positive fraction with biological activity as siderophore. HPLC purification of that fraction yielded a pure compound which was identified as citrate from its MS and NMR spectral data. Although the production of another citrate-based compound with siderophore activity cannot be ruled out, our results suggest that *Pdd* secretes endogenous citrate and use it for iron scavenging from the cell environment.

## Introduction

Iron restriction is an important host defense strategy, thus successful pathogens must possess mechanisms to acquire iron from host sources in order to cause disease. Although there are several strategies for iron acquisition, heme uptake and siderophore-based systems are certainly two of the most widespread among pathogenic bacteria and the most relevant to virulence (Hider and Kong, 2010; Cassat and Skaar, 2013; Saha et al., 2013; Ellermann and Arthur, 2017).

*Photobacterium damselae* is a member of *Vibrionaceae* that has been divided in two different subspecies: subsp. *damselae* (hereafter *Pdd*) and subsp. *piscicida* (*Pdp*). *Pdd* is an autochthonous marine bacterium resident in both natural and fish farms ecosystems that can behave as an opportunistic pathogen for fish and mammals (Labella et al., 2017). It is also a primary emerging pathogen causing haemorraghic septicemia in a variety of marine species including wild and cultured fish, sharks, crustaceans and marine mammals. In addition, *Pdd* can cause infections in humans due to exposure to marine fish, seawater or raw seafood (Rivas et al., 2013). In some of the reported human cases, the infection progressed into a severe necrotizing fasciitis leading to a fatal outcome (Yamane et al., 2004). In this bacterium it was demonstrated a positive correlation between iron availability in host fluids and degree of virulence (Fouz et al., 1997). Previous works proved that all *Pdd* strains are capable of utilizing hemoglobin and ferric ammonium citrate as the sole iron sources and that they also have the ability to produce siderophores (Fouz et al., 1994, 1997). It was postulated that the siderophore produced by *Pdd* could be a hydroxamate type different from aerobactin and desferal (Fouz et al., 1997), but the precise nature of the siderophores produced was not elucidated up to date.

In previous works, we described the presence of a functional heme uptake system in *Pdd* (Rio et al., 2005), but at present nothing is known about the siderophore-based systems used by *Pdd* to get iron from its hosts. In a recent work about *Pdd* proteome variations under diverse iron conditions (Puentes et al., 2017), we showed that some *Pdd* strains are likely able to produce the siderophore vibrioferrin, that was firstly described as the main siderophore of *Vibrio parahaemolyticus* and *V. alginolyticus* (Yamamoto et al., 1994). We showed that some *Pdd* strains express proteins with high similarity to the main proteins involved in the biosynthesis and transport of vibrioferrin in these vibrios (Puentes et al., 2017). However, many other pathogenic *Pdd* strains, including the reference strain CIP 102761 (ATCC 33539), do not express those proteins and lack the gene clusters involved in vibrioferrin production, but they still display a CAS reactivity, suggesting the existence of an alternative siderophore in these strains.

In this work we show that most pathogenic strains of *Pdd* secrete endogenous citrate and use it for iron scavenging from the cell environment.

## Materials and methods

### Strains, media, and reagents

*Pdd* strains were routinely grown at 25°C on Tryptone Soy Broth or Agar (Cultimed) supplemented with 1% NaCl (TSB-1 or TSA-1 respectively). Differential iron availability conditions were achieved by adding to CM9 minimal medium (Lemos et al., 1988) final concentrations of 10 μM Fe_2_(SO_4_)_3_ (high iron conditions), or different concentrations of the non-assimilable and non-toxic iron chelator 2,2′-dipyridyl (TCI): 40 μM to get low iron conditions or 75 μM for very low iron conditions. The addition of 2,2′-dipyridyl to culture media mimics the low iron conditions found in the host environment. Strains and plasmids used are listed in Table 1.

**Table 1:** Strains and plasmids used in this work.

**Strain or plasmid**	**Description**	**References/source**
**STRAIN**		
***Photobacterium damselae*** **subsp**. ***damselae***		
RM71	Isolated from turbot (see Table 3)	Laboratory stock
RM71-rif	RM71 derivative, spontaneous rifampin-resistant mutant; Rf^r^	Rivas et al., 2011
BP53	RM71 with mini-Tn*10* disrupting *idh* gene	This study
RM71Δ*gltA*	RM71 with in-frame deletion of *gltA* gene	This study
RG91	Isolated from turbot (see Table 3)	Laboratory stock
RG91Δ*pvsD*	RG91 with in-frame deletion of *pvsD* gene	Laboratory stock
***E. coli***		
DH5α	Cloning strain	Laboratory stock
S17-1 y *pir*	RP4 (Km::Tn7, Tc::Mu-1) *pro-82 *y* pir recA1 endA1 thiE1 hsdR17 creC510*	Herrero et al., 1990
**Plasmids**		
pLOFKm	Tn*10*-based delivery plasmid; Km^r^	Herrero et al., 1990
pKNG101	Suicide vector; St^r^	Kaniga et al., 1991
pUC118	High-copy-number cloning vector; Ap^r^	Vieira and Messing, 1987

### Growth under iron limiting conditions and test of siderophore production

To test the ability of *Pdd* strains to grow under iron limiting conditions, overnight cultures in LB were adjusted to an OD_600_ of 0.5 and diluted 1:100 in CM9 minimal medium containing the iron chelator 2,2′-dipyridyl at 40 or 75 μM. 2,2′-dipyridyl (TCI) was dissolved in distilled water to prepare a stock solution at 20 mM that was added to the sterile media at appropriated concentrations. When required, ferric-ammonium citrate (Panreac) was added to CM9 medium at 10 μM final concentration. Cultures were incubated at 25°C with shaking at 150 rpm, and OD_600_ was measured after 12 h of incubation. Siderophore production was measured using the colorimetric liquid assay of the Chrome-Azurol-S (CAS) dye, as previously described (Schwyn and Neilands, 1987; Balado et al., 2015, 2017b). For CAS-reactivity assays, strains were grown at 40 μM 2,2′-dipyridyl to allow enough growth to make iron-chelating activity detectable. A non-inoculated CM9 sample with the same 2,2′-dipyridyl concentration was used as spectrophotometric blank and as negative control for CAS liquid assays. Growth curves and CAS assays were carried out in triplicate, and results shown are the means of three independent experiments.

### Cross-feeding assays

Bioassays were designed to detect the production of vibrioferrin and to detect the production of other iron-chelating molecules. To detect vibrioferrin synthesis we used a mutant (AR13) of *Vibrio alginolyticus* deficient in *pvsA*, a gene involved in the synthesis of this siderophore (Osorio et al., 2015). For the detection of biological active supernatants we used strain RM71. The indicator strains were inoculated into CM9 plates containing the iron chelator 2,2′-dipyridyl at 100 μM, a concentration at which they are unable to growth. Strains to be tested were cultured on LB agar plates, cells were harvested with a sterile loop, placed on top of the indicator strain plates and incubated at 25°C for 48 h. The results were scored as positive when a growth halo of the indicator strains was visible around cells. A disc containing 10 μl of a solution of Fe_2_(SO_4_)_3_10 mM was used as positive growth control.

### DNA purification and analysis and PCR

Total genomic DNA from *Pdd* was purified with the Easy–DNA kit (Invitrogen). Plasmid DNA purification and extraction of DNA from agarose gels were carried out using kits from Fermentas (Thermo–Fisher). PCR reactions were routinely carried out in a T–Gradient Thermal Cycler (Biometra), with *Taq* polymerase KAPA Taq (Kapa Biosystems). Since all *Pdd* strains with genomic sequences available lack vibrioferrin genes, detection of *pvsD* gene by PCR was performed using degenerated oligonucleotides (Table 2) designed from conserved *pvsD* sequences from several *Vibrio* species aligned with the *ClustalW Multiple Alignment* tool of BioEdit program (http://www.mbio.ncsu.edu/BioEdit/bioedit.html). The choosen sequences were those of *pvsD* from *V. parahaemolyticus* (accession No: AB082123.1; GI: 23307114), *V. alginolyticus* (accession No: DQ201184.2; GI: 120564760), *V. splendidus* (accession No: NC_011744.2; GI: 294510242), *V. caribbenthicus* (accession No: EFP94847.1; GI: 309367283) and *V. harveyi* (accession No: EMR36193.1; GI: 471343428).

**Table 2:** Oligonucleotides used in this work for mutant construction and detection of *pvsD* gene by PCR.

**Oligonucleotide Sequences (5′–>3′)**	
***gltA*** **mutant construction**	
gltA-ISA-1	CCCCTGCAGGTCGACGGATCGAGCTTCTGTTTGAGCCAGC
gltA-ISA-2	ACATGAGAACCAAGGAGAATAGATTCACATGAGGCAGTAG
gltA-ISA-3	AGTACCTAGCCAAGGTGTGCGATCAACGTGAGTTTAGTCC
gltA-ISA-4	ACTTATGGTACCCGGGGATCTGAGTATAGCCGTCTGCAAG
**Degenerated primers for RG91** ***pvsD*** **amplification**	
pvsD_deg_F	CCCTTGYCAYCCTTGGGAAA
pvsD_deg_R	GAATCCADACRCARCABGGC
***Photobacterium damselae pvsD*** **PCR screening**	
pvsD-damsela_Fw	GCTTCACTGATGTTGTTGAT
pvsD-damsela_Rv	CGAGCAAAAAGAAGATCTGA

### Mutagenesis by mini-Tn*10* transposon

A mini-Tn*10* insertion library was constructed in *Pdd* strain RM71 using the conjugative suicide plasmid pLOF/Km, carrying a mini-Tn*10* transposon with an isopropyl-beta-D-thiogalactopyranoside (IPTG)-inducible transposase located outside the mobile element, and a kanamycin-resistant gene flanked by transposable elements (Herrero et al., 1990; Rivas et al., 2015). Conjugation of pLOF/Km into *Pdd* RM71 was achieved my mixing equal amounts of the *E. coli* S17(pLOF/Km) (previously cultured in LB at 37°C for 5 h) and RM71-Rif (rifampicin-resistant) (cultured in TSB-1 at 25°C for 12 h). One mL of the mixture plus 10 μL IPTG was deposited in Marine Agar (Difco) plates and incubated at 25°C for 72 h. Appropriate dilutions of the growing cells were then plated on LB agar containing kanamycin (50 μg/mL) and rifampicin (50 μg/mL) and incubated at 25°C for 48 h to select for *Pdd* transformants. An Km^R^ Rif^R^ clone was considered a mini-Tn*10* insertion mutant. Growth and CAS-reactivity for each mutant was tested in 96-wells microtiter plates containing 100 μL of CM9 with 25 μM 2,2′-dipyridyl. Sequencing of DNA fragments flanking transposon sequences in the clones with expected phenotypes was achieved by partial digestion of genomic DNA with restriction enzyme *BfuC*I (New England Biolabs) for 35 s. The restriction products were ligated into plasmid pUC118 previously treated with *BamH*I and alkaline phosphatase. The ligation products were transformed into *E. coli* DH5α, and clones were selected in LB with Km 50 μg/mL. pUC118-cloned inserts containing the kanamycin resistance gene of mini-Tn*10* plus flanking chromosomal DNA were purified using the GeneJET Plasmid Miniprep Kit (Thermo Fisher Scientific) and finally sequenced using a primer targeted to the extreme of the kanamycin cassette (TCCAGTTTACTTTGCAGGGC). DNA sequences were obtained using a capillary DNA Sequencer ABI 3730xl (Applied Biosystems), and then analyzed by BLAST (Altschul et al., 1990) to identify the insertion points.

### Allelic exchange mutagenesis

In-frame deletion of *gltA* gene in *Pdd* RM71 was constructed by allelic exchange using the nucleotide sequence of locus A0J47_03095, protein with accession No. ODA26781.1, from *Pdd* RM71 genome sequence. PCR amplifications of two fragments of the gene and flanking regions, when ligated together result in an in–frame (nonpolar) deletion. Primers used are listed in Table 2. The construction was ligated into the suicide vector pKNG101 (Kaniga et al., 1991). The resulting plasmids were mated from *E. coli* S17-1-λ*pir* (Herrero et al., 1990) into *Pdd* RM71 wild type strain (Rif^R^) and exconjugants with the plasmid (Streptomycin^R^) integrated in the chromosome by homologous recombination were selected. A second recombination event was obtained by selecting for sucrose (15%) resistance and further checking for plasmid loss and for allelic exchange, generating the mutant RM71Δ*gltA*. Deletion of the parental gene was checked by DNA sequencing of the region involved to ensure that mutation was in-frame.

### Citrate quantification

Bacterial cultures under high- and low-iron conditions in early exponential growth (OD_600_ = 0.6) were centrifuged (5 min at 8,000 × g) and filtered through 0.22 μm pore size to obtain a cell-free supernatant. The citrate concentration in these supernatants was measured using a colorimetric Citrate Assay Kit (Sigma-Aldrich) according to the manufacturer protocol. To remove oxaloacetate and pyruvate background a blank sample was included for each sample, omitting the Citrate Enzyme Mix provided by the kit, according to the manufacturer instructions. The citrate concentration standard curve was made with increasing concentrations (ranging from 0.1 to 12.5 mM) of sodium citrate (Merck) in CM9 medium. Concentrations of citrate lower than 0.1 mM gave reactions that were indistinguishable from CM9 alone. Three biological replicates were measured in duplicate.

### Isolation and identification of citrate from culture supernatants

*Pdd* strain RM71 was cultured in CM9 minimal medium supplemented with 35 μM 2,2′-dipyridyl. Cultures were carried out in 2 L Erlenmeyer flasks containing 1 L of medium. These flasks were inoculated with 20 mL of a fresh culture in TSB-1. Flasks were incubated during 24 h at 25°C with continuous shaking at 190 rpm. After incubation, bacterial cells were pelleted by centrifugation at 10,000 rpm in a Beckman J-21 high speed centrifuge. The supernatant was filtered through a continuous filtration cartridge with a 0.45 μm pore size membrane (Millipore) to remove any cell and organic debris from the supernatant. Siderophore activity present was evaluated by the CAS method as above. Equal volumes of supernatants and CAS solution were mixed and the absorbance at 630 nm was measured in a spectrophotometer after 20 min. Siderophore activity of fractions and purified compounds were monitored by bioassays as described above.

A 6-L batch of centrifuged cell-free culture broth was divided into five portions which were loaded onto a XAD-4 resin (Radius Column: 2.8 cm, resin mass: 120 g). After washing with distilled water (1.3 L) with a flow rate of 0.8 mL/min, the resin was eluted with a methanol/water (1:1) mixture followed by methanol. The siderophore and CAS activities remained in the fraction eluted with water which was then lyophilized to give 86.2 g of a solid. This solid was washed with MeOH (4 times × 290 mL) to yield, after removal of the solvent, 54.53 g of a siderophore/CAS active non-methanol-soluble white solid. The material was dissolved in H_2_O and chromatographed on a Sephadex LH-20 column which was eluted with a 9:1 mixture of H_2_O in MeOH at a flow rate of 1.8 mL/min. The collected fractions were submitted to CAS assay and the fractions of interest were concentrated under vacuum to afford a CAS-positive fraction (37.6 g). Part of that fraction (21.0 g) was divided in three batches and chromatographed on a Sephadex® G-25 Fine column which was eluted with 760 mL of H_2_O using a flow rate of 4.5 mL/min. The eluted fractions were submitted to CAS assay and a chloride test for the presence of salts. Fractions displaying an intense color change with the CAS reagent and with a relatively low amount of salts were pooled and lyophilized. The lyophilized material (400 mg) was dissolved in H_2_O and chromatographed on a Sephadex® G-10 column using H_2_O (87 mL) as eluent at a flow rate of 1.6 mL/min. Again, the eluted fractions were submitted to the same tests as before to afford 53.5 mg of a salt-free and CAS–positive fraction. Final purification of this fraction was achieved by HPLC using a Discovery® HS F5 (100 × 4.6 mm, 5 μm) column with a mobile phase consisting of an isocratic mixture of 0.3% of CH_3_CN in H_2_O (each containing 0.1% HCO_2_H) for 1.4 min and then, a gradient from 0.3 to 6.0% of CH_3_CN in H_2_O (each containing 0.1% HCO_2_H) over 15.8 min at a flow rate of 1.2 mL/min. The CAS-positive fractions, eluted with a retention time of 2.98 min, were pooled and concentrated *in vacuo* to provide 2.1 mg of compound **1** which was identified as citrate by its ^1^H NMR and (–)-ESIMS data. *Compound* **1**: ^1^H NMR (500 MHz in D_2_O) δ (ppm): 3.00 (2H, d, *J* = 15.7, 2H, 2-H), 2.84 (2H, d, *J* = 15.7, 2H, 4-H); (–)-ESIMS *m*/*z* 191 [M – H]^−^.

## Results

In order to identify the siderophore system in *Pdd*, two strategies were used simultaneously: (1) identification of genes that could be related to siderophore biosynthesis and uptake by screening a transposon-generated mutant collection; and (2) isolation of compounds with siderophore activity by HPLC fractionation of iron-deprived cell-free supernatants and further chemical characterization of the fractions that retained the biological activity.

### Only some *Pdd* strains harbor vibrioferrin biosynthetic genes

In previous works we have shown that some strains of *Pdd*, when cultured under iron limitation, express several proteins likely involved in the synthesis of the siderophore vibrioferrin (Puentes et al., 2017). In order to confirm the presence of vibrioferrin related genes, we checked by PCR the presence of *pvsD*, one of the main genes involved in the biosynthesis of this siderophore in other vibrios (Tanabe et al., 2003; Wang et al., 2007), in a collection of *Pdd* strains. The results are shown in Table 3. Some strains isolated from different hosts and different origins harbor the *pvsD* gene and give a strong reaction in the CAS test. Vibrioferrin production by these strains was assessed by a growth promotion bioassay using the *V. alginolyticus* AR13 mutant defective in vibrioferrin biosynthesis (Osorio et al., 2015). However, as seen in Table 3, there are many other virulent strains from different origins that clearly lack *pvsD* and do not produce vibrioferrin, but they still give a positive reaction in the CAS test for siderophore production, although with lower intensity.

**Table 3:** Results of the PCR assays to detect the presence of *pvsD* and results of the CAS test and bioassays to detect vibrioferrin production in a collection of *Pdd* strains.

**Strain**	**Origin**	**Host**	**Presence of *pvsD***	**CAS test^a^**	**Vibrioferrin Production^b^**
ATCC33539	USA	Damselfish (*Chromis punctipinnis*)	−	+	−
RG91	Spain	Turbot (*Psetta maxima*)	+	++	+
LD-07	Spain	Gilthead seabream (*Sparus aurata*)	−	+	−
RM71	Spain	Turbot (*Psetta maxima*)	−	+	−
RG214	Spain	Turbot (*Psetta maxima*)	+	++	+
CDC2227-81	USA	Human	−	+	−
ATCC35083	USA	Shark (*Carcharhinus plumbeus*)	−	+	−
ACR208.1	Spain	Turbot (*Psetta maxima*)	+	++	+
ACRp-72.1	Spain	Turbot (*Psetta maxima*)	−	+	−
TW294 L2	Spain	Seabass (*Dicentrarchus labrax*)	−	+	−
TW250/03	Spain	Gilthead seabream (*Sparus aurata*)	−	+	−
J3G-801	Taiwan	Shrimp (*Penaeus monodon*)	−	+	−
238	USA	Dolphin (*Tursiops truncatus*)	−	+	−
158	Belgium	Eel (*Anguilla anguilla*)	−	+	−
RG153	Spain	Turbot (*Psetta maxima*)	+	++	+
309	Spain	Mussel (*Mytilus edulis*)	−	+	−
PG-801	Taiwan	Shrimp (*Penaeus monodon*)	−	+	−
430	Spain	Seawater	−	+	−
H22060601R	Spain	Redbanded seabream (*Pagrus auriga*)	+	++	+
D20040408U	Spain	Gilthead seabream (*Sparus aurata*)	−	+	−
H011004020	Spain	Redbanded seabream (*Pagrus auriga*)	−	+	−
LB07070501R	Spain	Seabass (*Dicentrarchus labrax*)	−	+	−
S04070503C	Spain	Sargo (*Diplodus sargus*)	−	+	−
LCA24907	Spain	Barramundi perch (*Lates calcarifer*)	−	+	−
9FT1M-3	USA	Shark (*Carcharhinus* sp.)	−	+	−
RS80L1V1	USA	Red snapper (*Lutjanus campechanus*)	−	+	−
ST-1	USA	Trout	−	+	−
RS78SPL1	USA	Red snapper (*Lutjanus campechanus*)	−	+	−
9FT2B-2	USA	Shark (*Carcharhinus* sp.)	+	+	+

a *Reaction in CAS liquid test after growth in CM9 plus 40 μM 2,2′-dypiridyl; +, A_630_ < −0.6; ++, A_630_ ranging from −0.6 to −0.8*.

b *Detected by growth promotion of Vibrio alginolyticus AR13 (Osorio et al., 2015)*.

### Search of candidate genes encoding a siderophore system in vibrioferrin-negative strains

*Pdd* RM71 strain was chosen to decipher the putative siderophore-based iron uptake system present in *Pdd* strains lacking vibrioferrin system. This strain has been widely used in previous works of our research group to characterize diverse virulence factors (Rivas et al., 2011). To try to find candidate genes that could encode a siderophore-mediated iron acquisition system, we performed an *in silico* search on the genome sequence of strain RM71 (GenBank accession No. NZ_LYBT01000000) and on the reference genome from strain CIP 102761 (GenBank accession No. NZ_ADBS00000000). After this search, we were unable to find any ORF which could be potentially involved in siderophore synthesis or transport according to its homology to other siderophore-related genes.

Since *in silico* search gave negative results we performed a random mutagenesis approach in strain RM71, using the mini-Tn*10* transposon, to isolate mutants with siderophore production inactivated or altered. Essentially, we sought mutants that, compared to parental strain, showed a growth deficiency or a negative CAS reaction when they were grown under iron deficiency conditions. We also looked at potentially deregulated mutants that were positive in CAS assay under iron excess conditions. Any of these phenotypes could be indicative of inactivation of a component of a siderophore-mediated iron uptake system.

**Figure 1:**
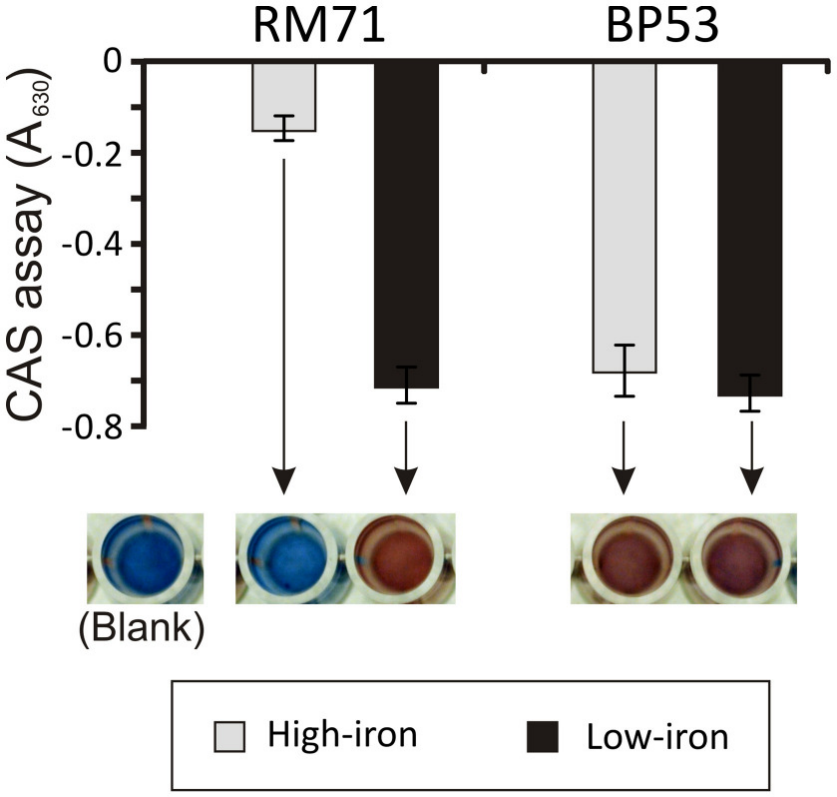
CAS assay of cell-free supernatants obtained from RM71 and BP53 mutant cultures at OD_600_ = 0.6 under high- and low-iron conditions. Each bar represents mean values from three replicates. Error bars indicate standard deviations. Color pictures show wells from a microtiter plate with representative CAS test results.

**Figure 2:**
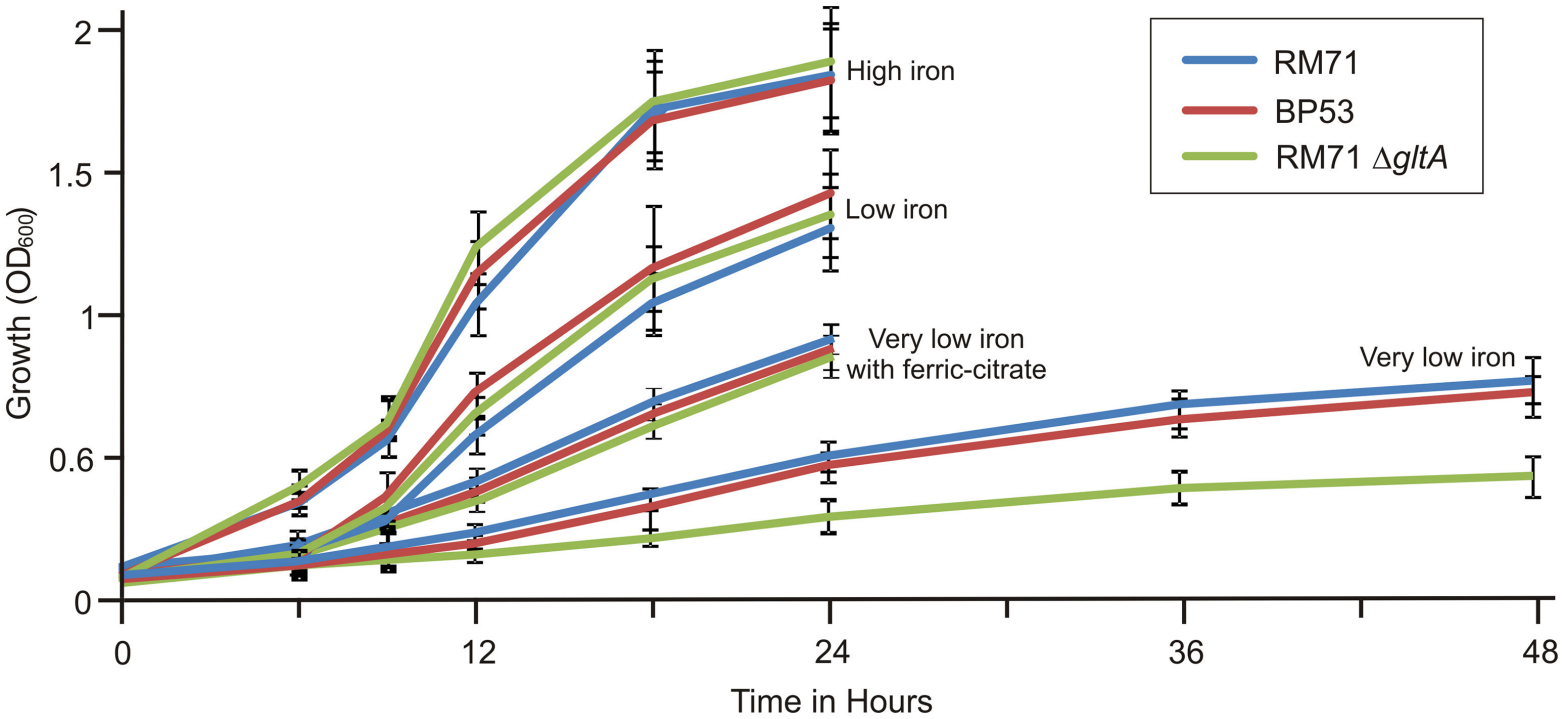
Growth curves of RM71 and mutants BP53 and RM71Δ*gltA* under different iron conditions. High iron: CM9 supplemented with 10 μM Fe_2_(SO_4_)_3_; low iron: CM9 plus 40 μM 2,2′-dipyridyl; very low iron: CM9 plus 75 μM 2,2′-dipyridyl. Each point represents mean values from three replicates. Error bars indicate standard deviations.

**Figure 3:**
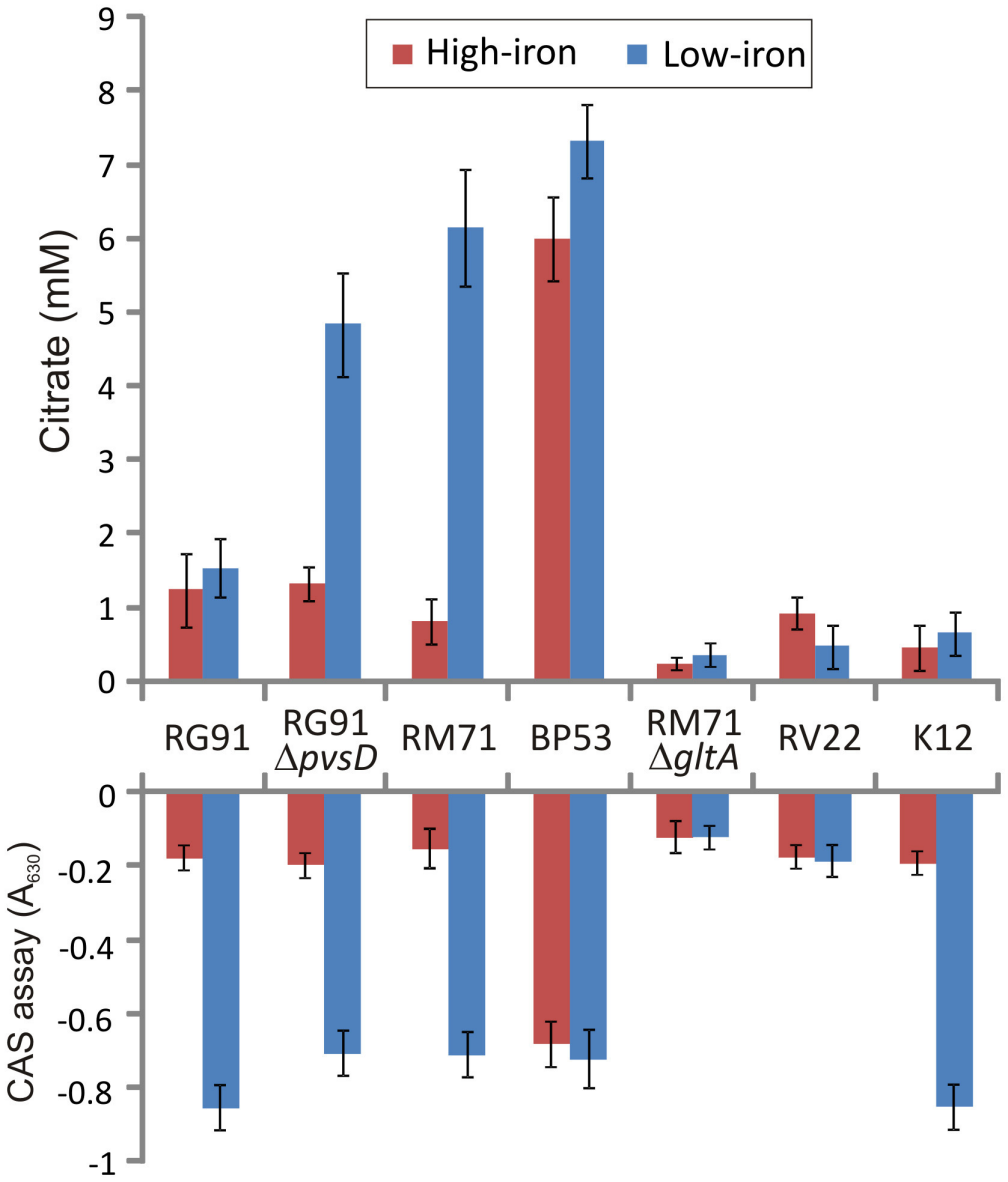
Extracelular citrate accumulation and CAS reactivity in cell free supernatants after growing *Pdd* strains under iron-excess or iron-deficient conditions. *Vibrio anguillarum* RV22 and *E. coli* K12 were used as controls. CAS reactivity of *E. coli* K12 is due to enterobactin production. Each bar represents mean values from three replicates. Error bars indicate standard deviations.

From a total of 1,400 mutants screened with the above phenotypic criteria we could found one mutant (BP53) that showed a strong CAS-positive reaction under both iron-deprivation and iron-excess conditions, which suggested an overproduction of iron-chelating compounds (Figure 1). Since the transposon library coverage was low (ca. 37%) we cannot discard the existence of other genes involved in iron uptake. Nucleotide sequence analysis revealed that mutant BP53 had the transposon inserted into *idh* gene (acc. No. ODA21722.1). This gene (named *icg* in *Vibrio cholerae*) encodes a NADP-dependent isocitrate dehydrogenase that participates in the tricarboxylic acid (TCA) cycle and convert D-isocitrate to 2-oxoglutarate with the production of one molecule of CO_2_. Inactivation of *icg* in *V. cholerae* and other bacteria results in an accumulation of large amounts of citrate and a slight growth impairment compared to its parental strain, especially during the late exponential phase of growth (Lakshmi and Helling, 1976; Minato et al., 2013). However, it is noticeable that BP53 did not show any growth defect in TSA-1 or CM9 media under high, low or very low iron (see methods) conditions (Figure 2). Furthermore, a significant citrate accumulation in the supernatant (ranging from 6 to 8 mM) could be detected (Figure 3). Based in these findings we could hypothesize that BP53 mutant secretes endogenous citrate to the extracellular medium and that the CAS-positive reaction observed could be caused by citrate accumulation.

### Extracellular citrate accumulation by *Pdd* strains

To confirm presence of citrate in culture media of *Pdd* strains we measured citrate levels under different culture conditions using a colorimetric citrate assay kit. We showed that RM71 produces noticeable levels of citrate, around 5.5 mM, when cultured in CM9 plus 40 μM 2,2′-dipyridyl, but under iron excess conditions the levels of extracellular citrate were under 1 mM (Figure 3). As CM9 does not include citrate in its formulation, the citrate present in the cell-free spent medium must come from bacterial cells synthesis. As comparative purposes we used *E. coli* K12 and *Vibrio anguillarum* RV22. The citrate amount present in these controls ranged from 0.8 to 1.3 mM and did not show any significant variation with iron availability. These data are in agreement with extracellular citrate amounts described for *E. coli* (Bennett et al., 2009). Thus, under iron-excess conditions the citrate amount found in RM71 supernatant (ca. 0.9 mM) is equivalent to the amount found in *E. coli* or *V. anguillarum*. Instead, citrate levels of RM71 increased 4- to 6-fold under low iron conditions. Hence, low levels of iron in the cell environment seem to trigger citrate synthesis and secretion in *Pdd* strains lacking the vibrioferrin system.

**Figure 4:**
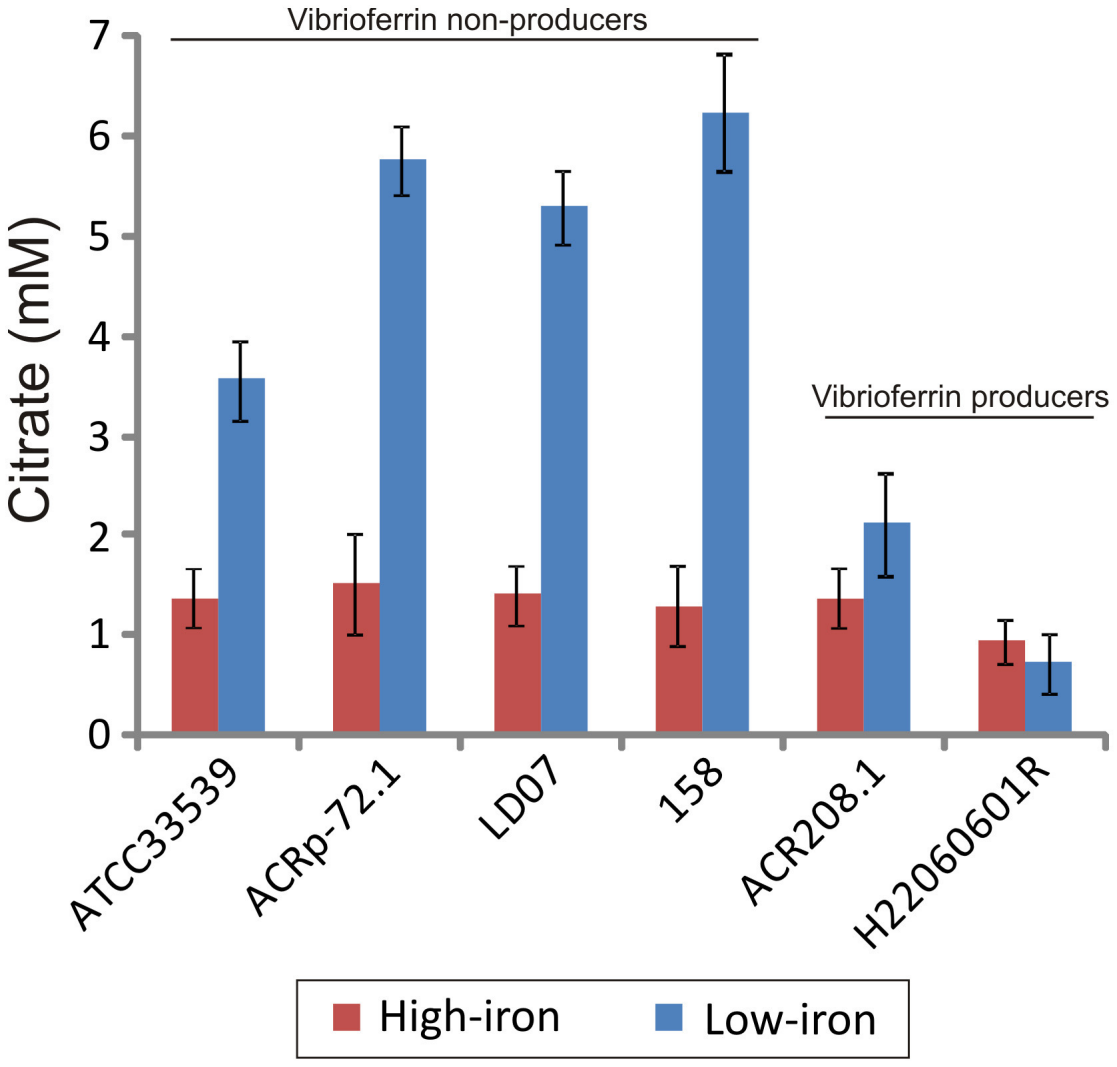
Citrate accumulation of several representative strains of *Pdd* producing and non-producing vibrioferrin. Each bar represents mean values from three replicates. Error bars indicate standard deviations.

Interestingly, when citrate accumulation was measured in a vibrioferrrin-producing strain (strain RG91, see Table 3), the citrate levels were 5- to 6-fold lower (ca. 1.5 mM) and independent of the iron levels of the medium (Figure 3). This observation could be explained by the hypothesis that if cells get enough iron through the likely much more efficient vibrioferrin system, citrate levels are kept in the minimal amounts needed for TCA cycle functionality. Only when a high affinity siderophore is not present, citrate would be synthesized at high levels to act as iron carrier for the cell. To confirm this hypothesis extracellular citrate levels were measured in a vibrioferrin-deficient mutant of RG91 lacking *pvsD* (RG91Δ*pvsD*). The results showed that in this mutant citrate accumulation reached the levels of strain RM71 and were also iron-dependent. In this mutant, the CAS-reactivity levels were also equivalent to those of RM71. Further evaluation of citrate accumulation in the supernatants of other *Pdd* strains, including vibrioferrin-producing and non-producing strains (Table 3), showed that the described behavior for each type of strain is not restricted to RG91or RM71 strains, but it seems a common feature in *Pdd* strains (Figure 4): citrate accumulates extracellularly only when vibrioferrin is not produced. All this suggests that citrate is being secreted by *Pdd* strains to the medium in high amounts when no other siderophore-based iron acquisition system is available.

To further confirm that citrate is involved in iron uptake in *Pdd*, a mutant lacking citrate synthase (GltA) was constructed in strain RM71 (RM71Δ*gltA*) and its phenotype was compared with parental strain RM71 and mutant BP53 (deficient in isocitrate deshidrogenase). All three strains showed identical growth curves under high iron conditions (Figure 2). When CM9 contained 40 μM 2,2′-dipyridyl (low-iron conditions, 50% of 2,2′-dipyridyl minimal inhibitory concentration, MIC) RM71, BP53, and RM71Δ*gltA* also showed indistinguishable growth curves (Figure 2). However, under very low-iron conditions (75 μM 2,2′-dipyridyl, 80% of MIC) RM71Δ*gltA* mutant showed a growth which was 45% lower with respect to BP53 and RM71. Interestingly, when ferric-ammonium citrate was added to the medium, RM71Δ*gltA* showed a growth curve identical to RM71 and BP53 strains (Figure 2). Therefore, RM71Δ*gltA* showed a significant decrease in the growth ability under iron deprivation conditions when the concentration of 2,2′-dipyridyl is close to the MIC of this iron chelator for the parental strain RM71.

### Citrate as siderophore for *Pdd*

The RM71 supernatant produces a CAS-positive reaction under low-iron, being the absorbance between 5- and 7-fold lower than in iron-excess conditions, which denotes that, as expected, siderophore activity is stronger under iron-deprived conditions. The iron homeostasis is strictly controlled by Fur repression, therefore the genes involved in iron uptake, like siderophore synthesis, are expressed only under iron starvation (Fillat, 2014; Porcheron and Dozois, 2015). As previously mentioned, BP53 showed a strong CAS-positive reaction under both iron-restricted and iron-excess conditions which denotes that *idh* inactivation causes an overproduction of siderophore. Interestingly, the supernatants from the RM71Δ*gltA* mutant showed a CAS-negative reaction in both high- and low-iron conditions (Figure 3) indicating that no detectable amount of siderophores was produced. Therefore, inactivation of citrate synthesis in RM71 abolishes siderophore production.

**Figure 5:**
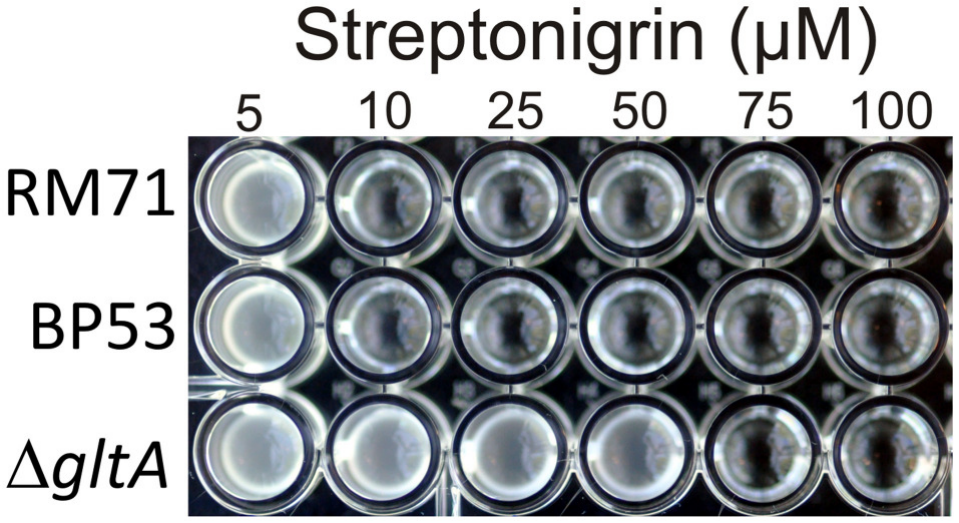
Growth of RM71 and mutants BP53 and RM71Δ*gltA* in presence of different concentrations (from 5 to 100 μM) of the iron-dependent antibiotic streptonigrin. Picture shows growth in a 96-well microtiter plate.

To further confirm that citrate serves as iron source for *Pdd*, we used the stretonigrin sensitivity method. Streptonigrin is a metal-dependent quinone-containing antibiotic which at high values of intracellular iron (Fe^2+^ and Fe^3+^) increases its bactericidal action by the formation of reactive oxygen radicals (Yeowell and White, 1982). So that, the presence in the cell of an active iron-uptake mechanism implies a growth defect when streptonigrin is added to the medium. In our case, while under iron limitation streptonigrin 10 μM was sufficient to inhibit the growth of RM71 and BP53 strains, the growth of RM71Δ*gltA* was inhibited only when streptonigrin was above 75 μM (Figure 5), indicating that no iron was entering into the cell. This result shows that blocking the endogenous citrate synthesis in *Pdd* RM71 prevents siderophore production, resulting in a decrease of growth under extreme iron deprivation. Altogether our results clearly suggest that citrate or a citrate-like compound serves as an iron scavenging system in RM71.

### Identification of CAS-reactive compounds in culture supernatants

**Figure 6:**
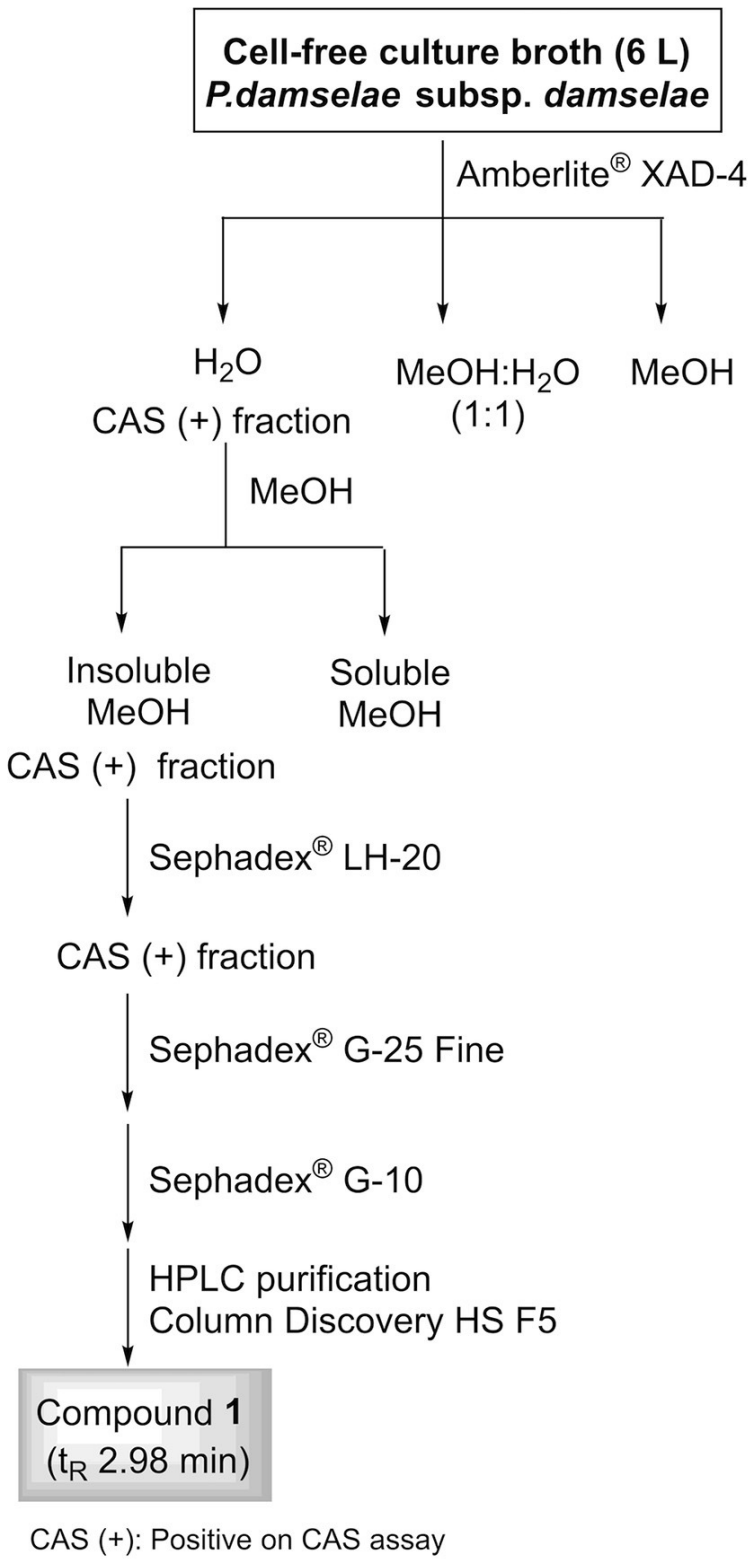
Fractionation flowchart and isolation of citrate (compound **1**) from the iron-restricted culture supernatants of *Pdd* strain RM71.

**Figure 7:**
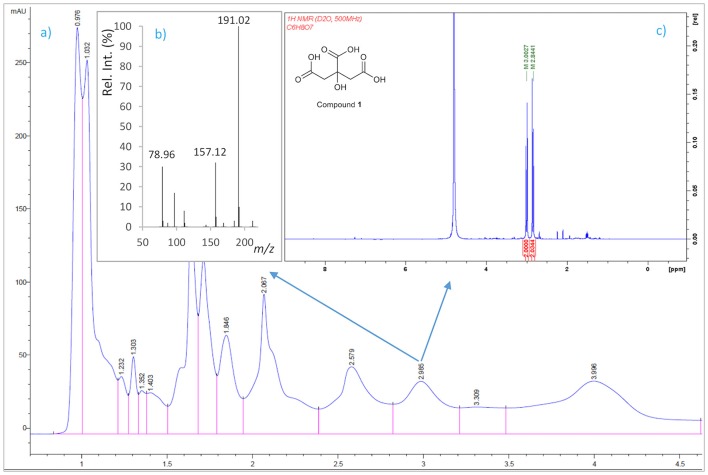
Isolation by HPLC and chemical analysis of compound **1**: **(a)** HPLC chromatogram of the CAS active and salt-free fraction eluted from the Sephadex® G-10 column. **(b)** (−)ESI mass spectrum and **(c)** ^1^H NMR spectrum in D_2_O (500 MHz) of compound **1**.

To identify compounds with siderophore activity secreted by strain RM71, the cells were grown under iron restricted conditions (CM9 with 35 μM 2,2′-dipyridyl) and the spent medium was subjected to chemical analysis using the CAS reactivity and biological activity as purification guides. The cell-free supernatants were fractionated by successive column chromatography (Amberlite XAD4 resin and size exclusion on Sephadex LH-20, G-25, and G-10) to give a CAS reactive and bioassay active fraction (Figure 6). From a final HPLC purification of this fraction we obtained a pure compound which spectral data (NMR, MS) were coincident to those of citrate (Figure 7). Hence, the CAS-reactive molecule produced by *Pdd* strain RM71 was unequivocally identified as citrate, and no other molecules with siderophore activity could be identified in the supernatants.

## Discussion

Citrate is produced by most microorganisms via the TCA cycle (Breusch, 1943) and, although it is not a powerful chelator, it binds Fe(III) forming a ferric-dicitrate complex (Silva et al., 2009). In this form, citrate can be used by many bacteria, including *Pdd*, as a source of iron (Pressler et al., 1988; Mazoy and Lemos, 1991; Fouz et al., 1994; Mazoy et al., 1997). Besides, citrate is a common moiety of many polycarboxilic siderophores (Hider and Kong, 2010).

Although, at neutral pH, citrate has lower affinity for iron than conventional siderophores, it is considered a high-affinity iron carrier, and it produces a CAS-positive reaction at concentrations above 0.1 mM (Frawley et al., 2013). Endogenous citrate secretion was described previously in other pathogenic bacteria and it is associated to intracellular iron homeostasis. However, while most bacteria can use externally supplied ferric citrate to fulfill their nutritional requirement for iron, there are two examples of bacteria which secrete citrate in order to get iron: *Pseudomonas syringae* (Jones and Wildermuth, 2011) and *Bradyrhizobium japonicum* (Guerinot et al., 1990). This property depends on the existence of a secretion system that secretes citrate under appropriate conditions. In *Salmonella typhimurium* it was described a major facilitator superfamily pump (IceT) that regulates intracellular iron content by Fe-citrate secretion (Frawley et al., 2013). *in silico* searches on *Pdd* genomes did not detect IceT homologs. Whether citrate is host or endogenous derived, bacterial cells require an energy-dependent transporter to acquire it, with the likely participation of outer membrane transporters from the TonB family (Ogierman and Braun, 2003). In *E. coli* and *Pseudomonas aeruginosa* this role is played by outer membrane protein FecA (Pressler et al., 1988; Marshall et al., 2009). Although up to five TonB-dependent transporters are present in *Pdd* genomes, a FecA homolog seems to be absent. However, a FecB homolog, a periplasmic transporter which is part of the ferric-citrate transport system in *E. coli* (Banerjee et al., 2016), has been previously identified as a protein expressed under low iron conditions in *Pdd* (Puentes et al., 2017). More work is needed to clarify the ferric-citrate transport in this bacterium.

Besides acting as iron chelator, citrate seems to play different roles. In *S. typhimurium* it was reported the existence of a stress-induced citrate secretion that would reduce the intracellular iron amount and arrest growth, increasing tolerance to antibiotics and to reactive oxygen species produced by the host defenses (Frawley et al., 2013).

It is known that TCA cycle is suppressed in anaerobiosis or in media containing glucose and that it is activated in presence of oxygen and acetate (Park et al., 1994). Fur is a negative regulator of iron acquisition systems that also exert a regulatory role in energy metabolism (Thompson et al., 2002). In addition, Fur regulates some TCA cycle steps. While citrate synthase is repressed by the Fe^2+^-Fur complex, the Aconitase A (that converts citrate into isocitrate) is induced by Fe^2+^-Fur, suggesting that some bacteria, like *E. coli*, respond to iron restriction by producing citrate (Thompson et al., 2002; McHugh et al., 2003).

Our results indicate that inactivation of TCA cycle observed in *Pdd* BP53 mutant results in a dramatic increase of citrate in the extracellular medium but it did not affect growth in glucose-containing media. These findings clearly suggest the necessary existence of a citrate secretion system that prevents intracellular citrate accumulation. Overproduction of citrate is deleterious to most bacteria. It was proved that cytoplasmic citrate accumulation causes a growth defect and promotes a strong selection pressure for mutations inactivating citrate synthase. Such mutations restore the growth potential in mutant strains that accumulate citrate. This was demonstrated for many microorganisms such as *E. coli* (Gruer et al., 1997), *Corynebacterium glutamicum* (Baumgart et al., 2011), *Sinorhizobium meliloti* (Koziol et al., 2009), *Vibrio fischeri* (Septer et al., 2015), and *Bacillus subtilis* (Pechter et al., 2013). Nevertheless, the accumulation of citrate seems to have no negative effect on *Pdd* growth, suggesting that most citrate excess must be secreted to the extracellular medium to avoid intracellular accumulation. In contrast, inactivation of citrate synthase in *Pdd* produces a significant decrease in growth capacity under extreme iron-restriction. Therefore, citrate secretion by *Pdd* has a positive effect on cell fitness and could be used for extracellular high-affinity iron scavenging, which suggests that *Pdd* likely synthesizes a citrate excess to use it for iron uptake from the cell surroundings. This is reinforced by the observation that a vibrioferrin-producing *Pdd* strain does not accumulate citrate, but when the vibrioferrin system is inactivacted, the mutant behaves like a strain lacking the vibrioferrin system, and accumulate extracellular citrate in an iron-dependent fashion (Figure 3). One plausible explanation of this result is that since citrate is a precursor for vibrioferrin (Tanabe et al., 2003), mutation of a vibrioferrin biosynthetic gene would result in the accumulation of precursors, and in this case citrate would be exported out of the cell to avoid toxicity. The cell would then use external citrate as an alternative iron carrier.

The advantages of using citrate or vibrioferrin for iron uptake are unclear. From the results described here we could deduce that vibrioferrin use would be a better strategy since all strains that produce this siderophore do not accumulate citrate. However, the vibrioferrin affinity constant for iron is not particularly high, with respect to citrate (Amin et al., 2009; Silva et al., 2009). Furthermore, and despite that the information available is scarce, seems that there are not clear differences in the virulence degree between the two types of strains (data not shown). In addition, it is likely that the use of citrate could be an ancestral mechanism to get iron since *Pdd* and *Pdp* subspecies share a common evolutionary origin (Balado et al., 2017a), and we have some evidence that *Pdp* seems to use also secreted citrate for iron uptake. Some strains of each subspecies could have later acquired by horizontal transfer additional siderophore-based mechanisms for iron uptake, like the plasmid-encoded piscibactin in *Pdp* (Osorio et al., 2015) or vibrioferrin in *Pdd*, resulting in the inactivation of the use of secreted citrate for iron uptake.

As conclusion, this work shows that although some strains of the marine pathogen *Pdd* produce vibrioferrin as siderophore, most pathogenic strains release endogenous citrate to the extracellular environment in response to iron deprivation, and that this trait have a positive effect in the cell fitness when it grows under extreme iron-restricted conditions. Although the production of another citrate-based or other compounds with siderophore activity cannot be completely discarded, our results suggest that endogenous citrate, besides being part of other siderophores, can be itself used for iron uptake by *Pdd*.

## Author contributions

MB, CO, CJ, and ML conceived and designed the experiments. BP, LC, MB, and JF performed the laboratory experiments. CO and JR made substantial contributions to conception and design. MB, LC, CJ, and ML participated in experimental design and data analysis. MB, CJ, and ML drafted and wrote the manuscript. All authors read and approved the final manuscript.

### Conflict of interest statement

The authors declare that the research was conducted in the absence of any commercial or financial relationships that could be construed as a potential conflict of interest.
